# Migraine, monoclonal antibodies, and monitoring: a review of pharmacovigilance in the era of biologics

**DOI:** 10.3389/fphar.2026.1822176

**Published:** 2026-05-29

**Authors:** Lorenza Guarnieri, Francesca Bosco, Maria Tiziana Corasaniti, Rita Citraro, Giovambattista De Sarro

**Affiliations:** 1 Section of Pharmacology, Department of Health Sciences, University “Magna Graecia” of Catanzaro, Catanzaro, Italy; 2 Research Center FAS@UMG, Department of Health Science, University “Magna Graecia” of Catanzaro, Catanzaro, Italy

**Keywords:** biologics, biosimilar, calcitonin gene-related peptide, migraine, pharmacovigilance

## Abstract

Pharmacovigilance practices are essential for the rational use of medicines; this is even more true in the field of biologics. An ideal pharmacovigilance activity should cover all life phases of a drug to obtain more information on efficacy and safety by overcoming the limitations of pre-marketing studies. Active pharmacovigilance carried out during the post-marketing phase aims to fill these gaps by using continuous and pre-organized processes to fully identify the number of adverse events. In recent years, the number of biologic drugs approved by the Food and Drug Administration (FDA) has increased greatly. In the last decade, there has been a growing interest in identifying monoclonal antibodies against Calcitonin Gene-Related Peptide (CGRP) for treating migraines. However, pharmacovigilance on these biologics suffers from a paucity of data, and the limitations of pre-marketing experience, the additional risks associated with the intrinsic characteristics of biologic compounds, and the chronic use of most of these drugs make intensive and continuous pharmacovigilance activity necessary. This review aims to bring together all data on the efficacy and safety of biologics used to treat migraine. Both pre-marketing clinical trials and real-world post-marketing studies will be examined to provide a comprehensive overview of the data in the literature.

## Graphical Abstract

**Figure:**
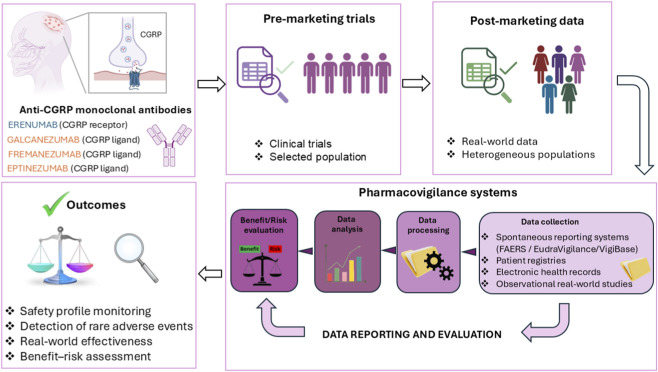

## 1 Pathophysiology of migraine and the CGRP pathway

Migraine is one of the most common and disabling neurological disorders, affecting more than one billion people worldwide, with a higher prevalence in women and a peak incidence between 25 and 55 years of age (Yeh et al., 2018; GBD 2017 Disease and Injury Incidence and Prevalence Collaborators, 2018; Ailani and Blumenfeld, 2022; Pezzini et al., 2010; GBD 2016 Headache Collaborators, 2018). It involves repeated attacks of moderate to severe, throbbing, side-localized headache, along with symptoms that may impair the performance of normal daily activities, such as feeling unwell, vomiting, and extreme sensitivity to light and sound (American Headache, 2019; Alpuente et al., 2022). It can go from occurring from time to time, when it is episodic migraine (EM), to becoming chronic migraine (CM), representing a constant problem. This dramatic evolution is observed in about 3% of patients every year (Aurora and Brin, 2017; Bigal et al., 2008a; Scher et al., 2003). Indeed, one aspect that makes this condition complicated is its worsening over time: migraine is not just a bad headache, but a real brain disorder that in the long run is associated with changes in the appearance and functioning of the brain (Bashir et al., 2013; Ashina S. et al., 2021).

CM, defined as a headache that occurs for at least 15 days per month (8 of which have migraine characteristics) for more than 3 months (Author Anonymous, 2018), impacts the quality of life (QoL) in 0.5%–5% of people (Bigal et al., 2008b; Natoli et al., 2010). It carries a heavy burden, causing greater disability, worse quality of life, and negative repercussions on work and social relationships than EM (Bigal et al., 2008b; Buse et al., 2012; Bigal et al., 2003).

In Italy, CM is often not properly diagnosed and treated (Lipton and Silberstein, 2015; Cevoli et al., 2009), although it has a high socioeconomic impact considering that more than half of patients experience severe functional disability and significant productivity losses (Steiner et al., 2014).

The onset and development of migraine are strongly influenced by environment, genetics, and lifestyle (Yeh et al., 2024).

Consequently, several factors can contribute to the transformation of EM into CM, including medication overuse, inadequate therapeutic response, and comorbid conditions (Katsarava et al., 2012; Ferrari et al., 2015).

People with CM often suffer from psychiatric comorbidities such as depression and anxiety (Buse et al., 2010). In recent years, new knowledge has been highlighted on the interrelationships between migraine and other neurological and non-neurological pathologies. Epilepsy, mood disorders, and irritable bowel syndrome are, for example, comorbid pathologies associated with migraine that share common pathophysiological mechanisms (Cuciureanu et al., 2024; Sances et al., 2009).

Treatment of migraine is therefore not simple, and although pain relief may be sufficient for EM, prevention is necessary for CM (Hepp et al., 2014; Hepp et al., 2015). The main problem is that many preventive drugs do not work well or have significant side effects (Reuter, 2018; Edvinsson et al., 2018a).

The European Headache Federation (EHF) defines migraines as “resistant” if three types of drugs fail and “refractory” if nothing works (Sacco et al., 2020; Martelletti et al., 2014).

The failure to identify effective treatment and the fact that migraine attacks severely impact patients’ QoL result in a significant waste of healthcare resources (Bloudek et al., 2012; Irimia et al., 2011). Despite advances in understanding migraine pathophysiology and the development of new treatments effective management of chronic and difficult-to-treat migraine remains limited (Bashir et al., 2013; Ashina S. et al., 2021; Hepp et al., 2015).

To date, numerous substances have been identified as playing an important role in causing migraines and can be classified into three groups:

- Those that act on G protein-coupled receptors on the cell surface: calcitonin gene-related peptide (CGRP), pituitary adenylate cyclase-activating polypeptide (PACAP), histamine, prostanoids, and, more recently, amylin, vasoactive intestinal polypeptide (VIP), and adrenomedullin (ADM) (Ashina et al., 2017; Pellesi et al., 2020; Ghanizada et al., 2021a; Ghanizada et al., 2020; Ghanizada et al., 2021b);
- Those that bypass the cell membrane by acting directly at the intracellular level, accumulating the second messenger cyclic adenosine monophosphate (cAMP) or cyclic guanosine monophosphate (cGMP) (Guo et al., 2014);
- Those that cause the opening of potassium channels (Al-Karagholi et al., 2019; Al-Karagholi et al., 2021).

The neuropeptides CGRP and PACAP are released during migraine attacks following activation of the trigeminal-vascular system and act as powerful vasodilators (Dux et al., 2022; Pavelic et al., 2022), contributing to pain signal transmission (Edvinsson et al., 2018b; Russo, 2015). The signaling pathways of these two neuropeptides appear to be independent, but their functions are similar, and their levels may serve as potential biomarkers when assessed prospectively (Tanaka et al., 2023).

In recent decades, substantial evidence has accumulated supporting the role of CGRP as a key mediator of migraine, making it a relevant therapeutic target (Charles, 2018). CGRP is released into circulation during an acute migraine attack, and its concentrations are persistently elevated in patients with chronic migraine (Cernuda-Morollon et al., 2013). It has also been reported that CGRP infusion can trigger a migraine attack in predisposed individuals (Ashina et al., 2013) and that its concentration does not normalize following the administration of opioid drugs but is reduced following the administration of triptans (Goadsby et al., 1990) or various small-molecule CGRP antagonists (Marcus et al., 2014; Voss et al., 2016).

The effect of this peptide can be explained by the fact that stimulation of its receptors increases the second messenger cAMP, which in turn activates protein kinase A, leading to phosphorylation and the consequent activation (opening) of potassium channels. This activation is responsible for the hyperpolarization of smooth muscle cells (Brayden, 2002) and the decrease in Ca^2+^ entry into the cell, resulting in vasodilation (Quast, 1996) and ultimately neuroinflammation responsible for migraine pain (Luckhoff and Busse, 1990).

### 1.1 Monoclonal antibodies anti-CGRP

Since CGRP was discovered in 1982, the study of its related mechanisms has led to the application of monoclonal antibodies in migraine prevention therapy (Arkless et al., 2019). To date, positive results have been confirmed for therapy with the antibodies Galcanezumab, Eptinezumab, and Fremanezumab, all of which target the peptide (Caronna et al., 2024), and with Erenumab, which targets the CGRP receptor (Sun et al., 2016). These treatments have proven to be very promising from the outset as they are associated with a reduction in migraine attacks and an overall response rate of approximately 50% (Tanaka et al., 2023).

These are synthetic IgG molecules, therefore characterized by their large size, and are administered parenterally (intravenously, subcutaneously, or intramuscularly) since oral administration is limited by gastrointestinal degradation and poor systemic absorption (Caronna et al., 2024).

The intravenous route allows for complete bioavailability and the possibility of administering larger volumes and doses than the intramuscular or subcutaneous route but requires administration in a hospital setting. In contrast, bioavailability is highly variable (between 50% and 100%) for intramuscular and subcutaneous administration, and high doses are not permitted given the solubility of IgG (∼100–150 mg/mL). However, these routes offer the advantage of self-administration, which improves treatment convenience and long-term adherence. The long half-life of anti-CGRP antibodies allows for monthly or quarterly dosing, resulting in good adherence to therapy, and their high affinity and specificity for their target reduce drug interactions and off-target toxicity (Kielbasa and Helton, 2019; Aditya and Rattan, 2023).

Like all monoclonal antibodies, they can be immunogenic, triggering the production of anti-drug antibodies (ADAs) (Gasparini et al., 2013), which are influenced by many factors such as the production process, doses and frequency of doses, antibody structure, route of administration, concomitant immunomodulatory therapies and certain patient characteristics such as immune status or certain genetic characteristics (Burstein et al., 2015).

## 2 Monoclonal antibodies and pharmacovigilance: the need for enhanced control

In order to maintain an effective and quality healthcare system, it is necessary to continuously monitor the safety of medicines from their design through to their daily clinical administration (Khan et al., 2024). Pharmacovigilance, defined as “the science and activities relating to the detection, assessment, understanding and prevention of adverse effects or any other medicine/vaccine possible drug-related problems” (Kalisch et al., 2025), plays a crucial role in this context, ensuring that the benefits of drugs outweigh the risks associated with their use. During the phase of preclinical studies, the focus is on identifying safe dosing and establishing key safety parameters for subsequent clinical phases. However, it is in phase IV, or post-marketing surveillance, that pharmacovigilance assumes strategic importance, as it bridges controlled clinical trials with real-world clinical practice. At this stage that broader and more realistic assessments of the drug’s long-term safety profile emerge, allowing the identification of adverse reactions that are rare or have not yet emerged during the experimental phases (Kothari et al., 2024).

Adverse drug reactions are frequent events, particularly with the introduction of new drugs. These reactions can occur in mild forms, but in some cases, they can evolve into serious events, such as hypersensitivity reactions, the onset of new disease, permanent disability, or even fatal outcomes (Pirmohamed and Park, 2003).

In the specific case of monoclonal antibodies, the need for surveillance is even more urgent. Their complex biological nature, immunogenic potential, chronic therapeutic indications, and recent but increasing use in the clinical setting mean that these drugs require closer and more structured monitoring. Consequently, it is imperative to adopt an efficient and well-structured system to continuously monitor and analyze the safety of the drugs. This is precisely where pharmacovigilance comes in (Lee et al., 2025).

Recent developments in analytical techniques, such as machine learning, together with the increasing availability of digitized health data, are facilitating the accurate assessment of the risk-benefit ratio of drugs in the context of daily clinical practice (Vergetis et al., 2021). In parallel, the introduction of innovative therapies such as those with monoclonals has created a need for more targeted and specialized pharmacovigilance systems. These new therapeutics are entering the market with increasing frequency, posing a new challenge for pharmacovigilance, which must keep evolving in this area (Malheiro et al., 2025). Within this evolving framework, particular attention is required to the immunogenic potential of biologic drugs, which represents a key aspect for their post-marketing safety evaluation. Therapeutic antibodies can induce anti-drug antibodies (ADAs), altering efficacy and toxicity. Even fully human antibodies may remain immunogenic due to idiotypes, post-translational modifications, or production impurities (Gecse et al., 2022). Current data are insufficient to define the real impact of ADAs on efficacy and safety (Pellesi et al., 2021; Gupta and Gaurkar, 2022). ADAs are generated through T cell-dependent or independent mechanisms: the latter usually produce transient, low-affinity ADAs, whereas T cell-dependent activation *via* MHC II and cytokines promotes durable, high-affinity IgG ADAs, plasma cells, and memory B cells (Singh, 2011; Krishna and Nadler, 2016). Neutralizing ADAs (NAbs) directly interfere with mAb-target binding, reducing efficacy and altering half-life, while non-neutralizing ADAs can still affect pharmacokinetics through immune complex formation and clearance (Krishna and Nadler, 2016; Dingman and Balu-Iyer, 2019). These mechanisms may cause altered pharmacokinetics, loss of efficacy, cross-reactivity, and hypersensitivity reactions (Liu, 2015; Lewis et al., 2019). Type I reactions include itching, flushing, dyspnea, rash, and hypertension; Type II reactions involve ADAs/immune complex effects on cell membranes; Type III reactions derive from immune complex deposition with complement activation and tissue damage; and Type IV delayed reactions are T cell-mediated and range from rash to severe syndromes such as Stevens-Johnson syndrome and toxic epidermal necrolysis (Leach et al., 2014; Isabwe et al., 2018). In Fremanezumab, two heavy-chain constant-region mutations were introduced to prevent cell-mediated and complement-dependent cytotoxicity (Armour et al., 1999; Cohen et al., 2021).

### 2.1 The role of pharmacovigilance systems and databases

The advent of targeted therapy has revolutionized classical pharmacology, and today, biologics represent the cornerstones of modern medicine. Biologics are inherently characterized by a higher degree of structural complexity and intrinsic variability associated with manufacturing processes, which may translate into safety and efficacy profiles that differ from those observed with small-molecule drugs. Consequently, post-marketing surveillance plays a pivotal role (Kothari et al., 2024).

Due to, these differences, pharmacovigilance applied to biologics is more complex. Traditional pharmacovigilance based on the monitoring of small-molecule drugs is, in some respects, unsuitable for monitoring the safety of biologics.

For biologics, which do not yet have a long history behind them, strict post-marketing surveillance is required to carry out a detailed assessment of adverse effects with a view to rationalizing treatment.

In fact, Directive 2010/84/EU included biologics in the list of medicines subject to additional monitoring, and the European Medicines Agency (EMA) and FDA have adopted new recommendations and guidelines for the pharmacovigilance of these medicines in recent years (Woo, 2014).

In the context of pharmacovigilance for biologics, Post-Authorisation Safety Studies (PASS) and Post-Authorisation Efficacy Studies (PAES) are fundamental tools for gathering additional evidence after a medicine has been placed on the market. PASS studies are designed to identify, characterize, or quantify potential or known risks associated with the use of the medicinal product in real-world conditions, including populations that are often underrepresented in registration studies, such as the elderly, patients with comorbidities, or users of multiple therapies. At the same time, PAES studies allow the efficacy of the drug to be evaluated and confirmed in everyday clinical practice, providing useful data for understanding the therapeutic response in more heterogeneous and less controlled contexts than pre-authorization clinical trials. Overall, PASS and PAES contribute synergistically to a dynamic and continuous assessment of the benefit/risk ratio of biologics, supporting informed regulatory decisions and ensuring a high level of public health protection (Almeida et al., 2025).

However, for studies to be designed, conducted, and interpreted effectively, it is essential to have robust information systems and reliable data. In this context, pharmacovigilance databases play a central role: they are the main source of information on adverse reactions encountered in real-world clinical practice and enable the identification of safety signals that can then be investigated further through targeted post-authorization studies. The integration of spontaneous reporting and structured observational studies, therefore, allows for a synergistic approach, in which pharmacovigilance databases not only support early risk detection but also provide the information substrate necessary for planning PASS and PAES studies of high scientific relevance. In this context, the quality and traceability of the data contained in the databases become crucial aspects for ensuring effective monitoring and proactive management of the safety of biologics (Bihan et al., 2020).

A valid pharmacovigilance database must contain essential minimum data regarding an identifiable patient, a suspected medicinal product, and an identifiable adverse event. For biologics, however, these requirements are not sufficient: it is also essential to have the trade name, batch number, and other product traceability information, which are indispensable for correctly attributing the adverse event to a specific medicine and distinguishing between different products belonging to the same therapeutic class (Vermeer et al., 2013).

A robust database must accurately record all patient characteristics (age, sex, clinical conditions, comorbidities, any concomitant treatments) and contextual details of the adverse event, including severity, outcome, timing relative to exposure, and any actions taken (discontinuation, dosage reduction, re-challenge, therapeutic switch or swap). Similarly, it must include information on the suspected medicine, such as therapeutic indication, dosage regimen, route of administration, and duration of treatment. An effective system must allow for the standardization of data using international vocabularies and classifications (e.g., MedDRA for adverse events, WHO-Drug for drug coding), ensuring comparability and interoperability between different information systems. Furthermore, a high-quality database must ensure validation mechanisms, duplicate management, error checking, and continuous updating, as well as guaranteeing traceability of changes and compliance with personal data protection regulations. Finally, it must support analytical capabilities that enable early signal detection, time trend analysis, and integration with other real-world data sources, thus creating a solid information base to guide regulatory decisions and proactive pharmacovigilance activities (Vermeer et al., 2013).

### 2.2 The future potential of biosimilars in migraine therapy

The term biosimilar refers to a biological drug that is very similar to a reference medicinal product already on the market and has all the relevant characteristics of the original biologicals. These are also highly complex molecules with high molecular weight, produced in transgenic cells or organisms (Heinemann et al., 2023). Each biosimilar is like a reference biologic sin terms of efficacy, safety, and quality, and its approval is based on an abbreviated procedure that demonstrates its high similarity to reference biologics. In fact, compared to reference biologics, biosimilars are based on the experience already gained with the reference drug (Mascarenhas-Melo et al., 2024). Biosimilars must not only be highly similar to the reference biologic product but must also demonstrate no clinically significant differences. Similarity means that each biosimilar must be characterized and evaluated both structurally and functionally, according to specific development approaches in clinical trials (Kirchhoff et al., 2017). Above all, the primary objective of comparative clinical trials is not to re-establish efficacy and safety, but to identify significant clinical differences between the biosimilar and the reference biological product (Markenson et al., 2017).

Currently, no biosimilar targeting the CGRP pathway has been approved for migraine prevention. Therefore, any discussion regarding anti-CGRP biosimilars remains prospective and should be interpreted in light of evidence derived from other therapeutic areas. To date, migraine therapy has undergone considerable progress thanks to the advent of anti-CGRP monoclonal antibodies which demonstrated remarkable therapeutic efficacy in improving migraine frequency and QoL in patients suffering from both episodic and chronic forms (Scuteri et al., 2019). However, these agents are currently available only as originator biologics. Evidence from other areas of disease, particularly rheumatology and gastroenterology, provides a robust framework for contextualizing the potential role of biosimilars. Biosimilars of anti-TNF agents, including infliximab and adalimumab, have consistently demonstrated comparable efficacy, safety, and immunogenicity to their reference products across multiple indications such as rheumatoid arthritis and inflammatory bowel disease (Smolen et al., 2018; Cohen et al., 2018). Importantly, the evidence base supporting biosimilar use extends beyond pre-approval comparability exercises. Randomized switching trials, such as the NOR-SWITCH study, have shown that switching from originator infliximab to its biosimilar is not associated with significant differences in efficacy, safety, or immunogenicity across indications (Jorgensen et al., 2017). In addition, long-term extension studies and real-world data have further confirmed the effectiveness and safety of both single and multiple switches in routine clinical practice (Scheinberg and Azevedo, 2019; Gisbert and Chaparro, 2021). These findings have supported regulatory decisions on indication extrapolation and facilitated the broader adoption of biosimilars in clinical practice. However, the extrapolation of these data to migraine requires caution, as migraine is characterized by a complex neurobiological substrate and significant interindividual variability in response to monoclonal antibodies. Therefore, the hypothetical introduction of biosimilars in migraine therapy represents an important scientific and regulatory Frontier. Biosimilars have revolutionized the clinical approach in several areas, expanding access to targeted therapies in healthcare systems with limited resources, enabling more sustainable management of chronic conditions, and promoting the adoption of more personalized treatment protocols (Kvien et al., 2022). In this scenario, the introduction of biosimilar drugs for migraine represents a new challenge. The approval of anti-CGRP biologics has opened a new phase in migraine prophylaxis, but at the same time has also led to an imbalance in access to treatment. Therefore, the introduction of anti-CGRP biosimilar drugs could represent a significant advantage by ensuring effective and accessible treatments (Haridas et al., 2024). To date, no anti-CGRP biosimilar has been approved for migraine prophylaxis, but the expiry of patents on first-generation drugs is expected to catalyze the development of potential biosimilars (Versijpt et al., 2025). Their introduction compared to the original biologics could offer substantial advantages in terms of pharmacoeconomic aspects, reducing treatment costs and making biologic therapies accessible in public healthcare settings with limited resources, having a significant impact in countries where migraine is underdiagnosed and undertreated due to financial constraints (Scuteri et al., 2022; Al Meslamani, 2024; Coppola et al., 2025). However, despite the potential benefits of introducing biosimilars for migraine, their approval is not without challenges.

From a regulatory perspective, both the European Medicines Agency (EMA) and the U.S. Food and Drug Administration (FDA) have established rigorous but partially distinct frameworks for biosimilar approval. The EMA has pioneered the biosimilar regulatory pathway and adopts a stepwise, totality of evidence approach, including extensive analytical characterization, functional studies, and confirmatory clinical data. Importantly, the EMA allows extrapolation of indications across different clinical settings, provided that similarity is adequately demonstrated and scientifically justified (EMA, 2014; Kurki et al., 2017). In contrast, while the FDA similarly requires a comprehensive comparability exercise, it introduces an additional regulatory designation of interchangeability, which is not formally defined within the EMA framework. To obtain interchangeability status in the United States, biosimilars must provide additional evidence, typically including switching studies, demonstrating that they can be expected to produce the same clinical result as the reference product in any given patient, without increased risk in terms of safety or diminished efficacy (Cohen et al., 2018), (FDA, 2019). These regulatory differences may have important implications for clinical practice and market uptake. In particular, the absence of a formal interchangeability designation in Europe contrasts with substitution policies in the United States, potentially leading to heterogeneous adoption patterns. In the context of migraine, these aspects may become particularly relevant given the chronic use of anti-CGRP monoclonal antibodies and the need for long-term treatment continuity. The issue of interchangeability of biosimilars is one of the main limiting factors. Although the EMA and FDA regulatory agencies have established precise regulations for the approval of biosimilars, the attribution of interchangeability remains complex, since the interchangeability of future anti-CGRP biosimilars is further complicated by individual variability in response to mAbs, which is also influenced by polymorphisms in the CGRP pathway (Scuteri et al., 2022).

Once the originator’s patent expires, switching to a biosimilar will therefore depend on proven clinical robustness and post-marketing surveillance (Iskit, 2025); (Barbier and Vulto, 2022). Pharmacovigilance will assume even greater importance in this context, with long-term surveillance activities essential for detecting rare adverse events in neurologically vulnerable populations. Lessons from existing biosimilars highlight the importance of traceability, risk management plans, and the generation of Real-World evidence.

Therefore, clinical evidence will be crucial to validate the therapeutic efficacy and safety profile of biosimilars, guiding clinical decision-making (Chhabra et al., 2022; Liu et al., 2025). In conclusion, although biosimilars are not yet a concrete reality in migraine therapy, their possible integration could be plausible and promising, becoming a key pillar for expanding access to CGRP-targeted treatments.

### 2.3 Limitations of pre-marketing studies on anti-CGRP monoclonal antibodies

Pre-marketing clinical trials on monoclonal antibodies targeting CGRP were a crucial step towards the approval of this new class of drugs for migraine prophylaxis. However, these trials have several methodological and structural limitations that must be carefully considered when evaluating the safety and efficacy profile of these therapies (Ozge et al., 2024). Randomized clinical trials are conducted in highly controlled settings and typically enroll selected populations that differ substantially from real-world patients, reducing the external validity of the findings (Rothwell, 2005; Friede and n, 2017). A major limitation concerns patient selection. Clinical trials on anti-CGRP monoclonal antibodies systematically excluded subjects with cardiovascular, metabolic, or psychiatric comorbidities, as well as pregnant or breastfeeding women and pediatric patients. Although this strategy improves sample homogeneity and minimizes confounding factors, it limits the applicability of results to routine clinical practice, where migraine is frequently associated with multiple concomitant conditions (Van Der Arend et al., 2024; Tepper et al., 2017; Buse et al., 2019; Ashina et al., 2021a). Consequently, the information obtained in the pre-marketing phase cannot be considered exhaustive for assessing the safety of the drug in vulnerable or complex populations. Another limitation is the relatively short duration of prior studies. Most trials on anti-CGRP antibodies lasted between 12 and 24 weeks, which is sufficient to demonstrate efficacy in reducing attack frequency, but wholly insufficient to evaluate rare adverse events or side effects associated with chronic use (Goadsby et al., 2017; Silberstein et al., 2017). For example, the trial by Goadsby et al., which evaluated the effect of Erenumab for 12 weeks, confirmed the efficacy of the treatment but acknowledged the impossibility of identifying rare adverse events in such a short period of time (Goadsby et al., 2017). Similarly, the study by Silberstein et al. on Fremanezumab, also lasting 12 weeks, showed positive results but highlighted the need for longer follow-ups to define the safety profile (Silberstein et al., 2017). Even trials extended up to 24 weeks concluded that this duration was not sufficient to evaluate the long-term effects of Galcanebumab, making open-label extension studies indispensable (Martin et al., 2020). Migraine is a long-term condition, and anti-CGRP drugs are administered for years; therefore, safety assessment requires much longer follow-up (Lipton et al., 2007). Subsequent observational studies have in fact highlighted delayed responses and changes in the tolerability profile beyond 12 weeks, confirming the need for continuous monitoring (Barbanti et al., 2023; Obach et al., 2023). In this regard, the real-world study by Barbanti et al. made a particularly significant contribution: the authors followed patients treated with Erenumab for up to 12 months, documenting how a significant proportion of subjects showed progressive clinical improvement after the third month of therapy, with late responses that would not have been detectable in pre-marketing trials. Furthermore, the study highlighted the emergence of certain adverse events only during prolonged follow-up, suggesting that the tolerability profile may evolve and require continuous monitoring (Barbanti et al., 2021). Similar results were also reported by Obach et al., who evaluated the efficacy and safety of anti-CGRP antibodies in a large observational cohort in a real-world clinical setting. Again, extended follow-up allowed the identification of both late clinical responses and changes in the safety profile that had not emerged in trials of limited duration. In particular, the authors observed that some adverse events, although not serious, tended to occur after several months of treatment, confirming that safety assessment requires longer observation periods than the 12–24 weeks typical of registration studies (Obach et al., 2023). This limitation is consistent with what is known for other classes of monoclonal antibodies, for which rare adverse events often emerge only after marketing. This phenomenon has also been widely documented in other experiences with biologics, where it has been shown that numerous immune-mediated reactions associated with monoclonal antibodies only become recognizable through post-marketing pharmacovigilance, and that pre-marketing trials do not have sufficient statistical power to identify rare adverse events, making extensive monitoring in the real population essential (Pichler, 2006; Hansel et al., 2010). Although the sample size is large compared to many other trials, it remains limited when considering the need to identify low-incidence adverse events. Even when enrolling thousands of patients, the probability of detecting rare complications, such as cardiovascular or immunological events, remains very low. Only large-scale use in the general population can reveal such issues, once again highlighting the importance of post-marketing pharmacovigilance (Rikos et al., 2024). This concept is well known in clinical pharmacology, where events with an incidence of <1/10,000 cannot be identified in pre-marketing trials (Onakpoya, 2018). A relevant methodological aspect is the focus of endpoints on efficacy outcomes. Pre-marketing studies were primarily designed to demonstrate a reduction in the frequency of migraine attacks and an improvement in quality of life. Safety endpoints, although present, were not always explored in depth or structured to identify rare or late adverse events. Safety assessment focused on general parameters, such as the frequency of common adverse events or the percentage of treatment discontinuations, without detailed analysis of specific organs or systems (Van Der Arend et al., 2024; Ashina et al., 2021b). This approach is also evident in other pre-marketing studies on anti-CGRP antibodies. For example, in the phase 2 trial conducted by Dodick et al. on Erenumab, safety was assessed primarily through the frequency of common adverse events, without organ-specific analysis or in-depth monitoring of potential late effects (Dodick et al., 2018a). A similar approach can also be found in the PROMISE 1 trial on Eptinezumab, in which safety endpoints were limited to general parameters such as the overall frequency of adverse events and treatment discontinuations, without detailed exploration of possible rare or long-term effects (Ashina et al., 2022). Overall, these studies confirm that pre-marketing trials, while essential for demonstrating efficacy, are not designed to identify low-incidence or late adverse events, contributing to post-marketing observational studies indispensable (Caronna et al., 2024). The scientific literature confirms that safety endpoints in registration trials are often insufficient for a comprehensive assessment (Ioannidis, 2016). Important evidence gaps also persist for special populations. Robust data on pediatric patients, elderly subjects, and pregnant or breastfeeding women remain limited despite the clinical relevance of migraine in these groups (Oskoui et al., 2019; Noseda et al., 2021; Pons-Fuster et al., 2024; Mahadevan et al., 2013). Consequently, the safe extension of anti-CGRP therapies to these populations still requires dedicated studies and extensive post-marketing surveillance.

These limitations become particularly relevant in the context of future biosimilars. Although biosimilars for anti-CGRP monoclonal antibodies are not yet available, experience with other biologics such as anti-TNF and anti-IL agents demonstrates that biosimilar adoption can rapidly expand after market introduction (Machado et al., 2024). In such contexts, the scarcity of pre-marketing data on biosimilars, combined with the need to confirm comparability in terms of efficacy and safety, makes the role of pharmacovigilance even more crucial. The lack of extensive trials and reliance on limited equivalence studies does not allow clinically relevant differences to be completely ruled out, especially in complex populations or chronic treatments (Kohl, 2022). Therefore, experience with biosimilars could reinforce the idea that pre-marketing data, although indispensable, cannot alone guarantee a complete assessment of the risk profile, and that only intensive post-marketing monitoring can fill these gaps (Ebbers et al., 2012). In conclusion, pre-marketing studies on anti-CGRP monoclonal antibodies have provided valuable but partial information. Their controlled and limited nature does not allow us to fully grasp the complexity of real clinical practice, nor to anticipate the challenges that will arise with the introduction of biosimilars. For this reason, post-marketing pharmacovigilance and large-scale observational studies are indispensable tools for completing the picture and ensuring the safe and rational use of these drugs, both for biologics and for future biosimilars (Edwards and Aronson, 2000; Weise et al., 2014; Ingrasciott et al., 2018).

### 2.4 Management of adverse reactions and treatment discontinuations in anti-CGRP therapy

The management of adverse drug reactions (ADRs) and treatment discontinuations is another crucial aspect in assessing the safety profile of anti-CGRP monoclonal antibodies, especially in a context such as migraine, where preventive therapy is administered for prolonged periods and to patients often suffering from comorbidities (Van Der Arend et al., 2024). Pre-marketing studies have provided an initial reassuring picture, showing good overall tolerability, but their limited duration, participant selection, and systematic exclusion of complex populations (the elderly, frail patients, individuals with cardiovascular or gastrointestinal diseases) do not allow for a full understanding of the variability of real-world clinical practice (Ingrasciott et al., 2018). For this reason, post-marketing pharmacovigilance and large-scale observational studies are playing an increasingly central role in delineating the long-term safety profile of these therapies.

A recent meta-analysis conducted by the European Headache Federation on Erenumab, Galcanezumab, Fremanezumab and Eptinezumab integrated data from clinical trials and real-world studies, highlighting that, despite an overall favorable safety profile, treatment discontinuation may occur because of perceived lack of efficacy, ADRs, or patient-related factors. The most frequently reported Treatment-Related Adverse Events **(**TRAEs) include injection site reactions, gastrointestinal disorders, flu-like symptoms. Serious adverse events (SAEs) were uncommon, although cardiovascular and hypersensitivity events required closer clinical evaluation. The meta-analysis also highlights that the frequency and type of ADRs differ between controlled studies and clinical practice, suggesting that pre-marketing data, while indispensable, are not sufficient to comprehensively define the risk profile (Hoehne et al., 2026). Longitudinal observational studies have added further complexity. A large Italian cohort analyzed the effect of discontinuing anti-CGRP antibodies after 1 year of treatment, documenting that a significant percentage of patients discontinue therapy due to perceived ineffectiveness or the onset of clinically relevant ADRs (Vernieri et al., 2021). In this context, Erenumab emerges as the drug most frequently associated with gastrointestinal disorders, particularly constipation, which in some cases can be severe and refractory to conventional treatments. Increases in blood pressure have also been reported, sometimes to the extent that treatment had to be discontinued (Van Der Arend et al., 2024; Hoehne et al., 2026). For Galcanezumab and Fremanezumab, on the other hand, the most common ADRs include local reactions, skin rashes, and flu-like symptoms, which, although generally non serious, but could nevertheless contribute to treatment discontinuation (Hoehne et al., 2026). Eptinezumab, administered intravenously, has a different ADR profile, with infusion reactions and hypersensitivity being among the most frequently reported TRAEs. Most of these events were mild to moderate in severity; however, a smaller proportion were classified as serious adverse events (SAEs) or led to treatment discontinuation (Vernieri et al., 2021). Another prospective study analyzed clinical outcomes, unmet needs, and challenges in the management of patients who discontinue anti-CGRP antibodies, highlighting that discontinuations are not isolated events but an integral part of the therapeutic pathway. Treatments discontinuation rates vary but are often between 15% and 30% within the first year, with reasons ranging from perceived ineffectiveness to the onset of ADRs, to logistical factors or patient preferences. Importantly, not all ADRs resulted in treatment discontinuation, and only a subset of adverse reactions were considered clinically severe enough to require interruption of therapy. The study also showed that some patients request reintroduction of treatment after discontinuation, suggesting that active management of ADRs, for example, through treatment of constipation, blood pressure monitoring, or change of molecule, can significantly influence treatment persistence (Burgalassi et al., 2024). Overall, the available evidence indicates that the management of ADRs and treatment discontinuations requires a multidimensional approach, including a baseline risk assessment (cardiovascular and gastrointestinal comorbidities, concomitant therapies), periodic clinical monitoring (blood pressure, intestinal function, infusion reactions) and the use of patient-reported outcome tools to detect early signs of reduced tolerability. Real-world data and multicenter registries are therefore indispensable tools for supplementing the information provided by pre-marketing trials and ensuring the rational and safe use of anti-CGRP antibodies in everyday clinical practice (Barbanti et al., 2025). In addition, distinguishing between overall ADRs incidence, SAEs, and discontinuations due to ADRs is essential for an accurate interpretation of the safety profile of anti-CGRP monoclonal antibodies in clinical practice. Only an integrated approach allows for early identification of risk signals and the adoption of management strategies such as treatment of constipation, blood pressure monitoring, change of molecules, or temporary suspension with possible rechallenge. In conclusion, although anti-CGRP antibodies have revolutionized migraine prophylaxis, their safety profile must be continuously updated through active monitoring of ADRs and treatment discontinuations (Grazzi et al., 2024). The availability of real-world data and multicenter registries is essential to supplement the information provided by pre-marketing trials and to ensure the rational and safe use of these biological therapies (Yang et al., 2025).

## 3 Pharmacovigilance evidence on anti-CGRP monoclonal antibodies

In 2017, before the first anti-CGRP monoclonal antibodies for the treatment of migraine were approved, in a study examining the evidence available at the time, the authors reassured readers about their efficacy and safety, concluding that these drugs would represent more than just hope for millions of chronic migraine sufferers still without prophylactic therapy (Pellesi et al., 2017).

The first real-world studies on mAbs for migraine began to appear shortly after their approval in 2018, demonstrating their effectiveness in reducing monthly migraine days and improving QoL in clinical practice. Since the beginning, these studies have confirmed the efficacy of anti-CGRP mAbs in a larger and more diverse patient population than that examined in clinical trials, extending the observation to more diverse and complex patient populations, often including those with comorbidities or previous treatment failures.

Erenumab was the first therapeutic antibody approved by the FDA against the calcitonin gene-related peptide receptor (CGRP-R) (King et al., 2019).

### 3.1 Erenumab

One of the key studies for obtaining regulatory approval for this mAb was the LIBERTY study, whose data were essential in the pre-registration clinical development program, providing information on efficacy in patients with difficult-to-treat episodic migraine and on safety and tolerability in a complex population. This randomized, double-blind, placebo-controlled clinical trial lasted 12 weeks and was conducted in 59 centers in 16 countries, involving 246 patients aged between 18 and 65. The 30% of patients treated with Erenumab achieved the primary endpoint, i.e., a percentage of patients with a ≥50% reduction in monthly migraine days between weeks 9–12, compared with 14% in the placebo group. The safety profile was similar in both groups, and the most common adverse event was pain at the injection site (6%) (Tables 1, 2) (Reuter et al., 2018).

**TABLE 1:** Efficacy and safety evidence of Erenumab. Summary of the main available evidence on the efficacy and safety of Erenumab from both clinical trials and real-world studies.

Study	Population	Design/Duration	Efficacy	Safety	Key notes	Ref.
LIBERTY (RCT)	Difficult-to-treat EM (n = 246)	Randomized, double-blind vs. placebo; 12 weeks	≥50% reduction in MMD: 30% vs. 14% (placebo)	Similar to placebo; injection site pain (6%)	Pivotal registration trial	Reuter et al. (2018)
RWE – Brigham and Women’s Hospital (2020)	>400 patients (241 treated)	Retrospective	∼70% reported benefits > disadvantages; >60% willing to continue	∼70% reported ≥1 AE: constipation, fatigue, injection site reactions, worsening headache	Lower tolerability vs. trials; cost relevant	Kanaan et al. (2020)
RWE (n = 101)	Migraine (mostly chronic, heavily pretreated)	Post-marketing observational study	↓ headache days (−6.5); ↓ migraine days (−8.4)	28% discontinuation; constipation common; “wearing-off” effect	Complex population	Robblee et al. (2020)
EARLY 2 (Italy)	HFEM + CM (n = 221), ≥3 prior failures	Multicenter RWE; up to 48 weeks	≥50% response: 75.6% (CM), 56.1% (HFEM); ↓ analgesic use; improved QoL	Good tolerability	Dose escalation to 140 mg often required	Barbanti et al. (2021)
MAGIC (Canada)	EM + CM, ≥2 prior failures	Prospective observational; 24 weeks	∼33% achieved ≥50% response at 3 months; stable at 24 weeks	Well tolerated	Better outcomes in EM; physician + patient assessment	Becker et al. (2022)
Systematic review RWE (2024)	2,427 patients (12 studies)	Review of prospective cohorts	↓ MMD and MHD; ↓ acute medication use; improved QoL	Mostly mild AEs; low discontinuation	Sustained response; CM → EM conversion frequent	Nisar et al. (2024)
APOLLON (long-term)	EM + CM (n = 701)	Multicenter; 128 weeks	Sustained efficacy over time	Discontinuation due to AEs: 4.1%; no new safety signals	Discontinuation → worsening; re-initiation → rapid recovery	Gobel et al. (2024)

**TABLE 2:** Randomized controlled trials of anti-CGRP monoclonal antibodies in migraine prevention. Summary of the main randomized controlled trials evaluating the efficacy and safety of anti-CGRP monoclonal antibodies for migraine prevention.

mAb	Study	Population	Design/Duration	Efficacy	Safety	Key notes	Ref.
Erenumab	LIBERTY (Phase 3 RCT)	Difficult-to-treat episodic migraine (n = 246)	Randomized, double-blind vs. placebo; 12 weeks	≥50% MMD reduction: 30% vs. 14% (placebo)	Similar to placebo; injection site pain (6%)	Pivotal trial in treatment-refractory EM	Reuter et al. (2018)
Galcanezumab	EVOLVE-1/2 (Phase 3 RCTs)	Episodic migraine (>1,700 patients)	Randomized, double-blind, placebo-controlled	∼45–50% MMD reduction; consistent across subtypes	Mostly mild AEs; <5%; no serious treatment-related AEs	120 mg vs. 240 mg similar efficacy	Silberstein et al. (2019)
Galcanezumab	REGAIN (Phase 3 RCT)	Chronic migraine	Randomized, double-blind; 3 months + extension	Significant MMD reduction; sustained benefit; ↓ acute medication use	No relevant safety concerns	Efficacy independent of prior failures	Detke et al. (2018)
Fremanezumab	Phase 3 RCT (episodic migraine)	Episodic migraine (n = 875)	Randomized, double-blind; 12 weeks	MMD reduction: –1.5 days (monthly), −1.3 (single dose)	Injection site reactions; ∼2% discontinuation	First pivotal efficacy evidence	Dodick et al. (2018b)
Fremanezumab	Phase 3b RCT (treatment-resistant)	EM/CM with 2–4 prior failures (n = 838)	Randomized, double-blind; monthly vs. quarterly	MMD reduction vs. placebo (−3.7 to −4.1 vs. −0.6)	Serious AEs ∼1%; overall similar across groups	Flexible dosing (monthly/quarterly)	Ferrari et al. (2019)
Eptinezumab	PROMISE program (Phase 3 RCTs)	Episodic and chronic migraine	Randomized, double-blind	Significant MMD reduction with 100/300 mg	TEAEs mostly mild/moderate	IV administration → rapid onset	Ashina et al. (2020)
Eptinezumab	PROMISE-2 (Phase 3 RCT)	Chronic migraine (n = 1,072)	Randomized, double-blind; IV dosing; 24 weeks	Significant MMD reduction; ↑ ≥50% and ≥75% responders	No new safety signals	Sustained effect with repeated dosing	Lipto et al. (2020)

Later, in a retrospective real-world study conducted at Brigham and Women’s Hospital in Boston and published in 2020, patients treated with Erenumab at a tertiary headache center were evaluated. Through structured clinical interviews and review of medical records, data were collected from over 400 patients, 241 of whom had taken the drug. The results confirmed the effectiveness of the treatment: approximately 70% of patients reported that the benefits outweighed the disadvantages, and over 60% intended to continue with Erenumab. However, tolerability was also more problematic than reported in trials: as many as 70% of patients reported at least one side effect, with constipation, injection site reactions, fatigue, and worsening headaches among the most common. The cost of the drug was another critical factor, cited by some patients as a reason for discontinuing treatment (Tables 1, 3) (Kanaan et al., 2020).

**TABLE 3:** Real-world evidence of anti-CGRP monoclonal antibodies in migraine prevention. Summary of the main clinical and real-world studies evaluating the efficacy and safety profile of Fremanezumab in patients with episodic and chronic migraine.

mAb	Study	Population	Design/Duration	Efficacy	Safety	Key notes	Ref.
Erenumab	Brigham and Women’s hospitalRWE	>400 patients (241 treated)	Retrospective real-world study	∼70% reported benefit; >60% continuation	∼70% ≥ 1 AE (constipation, fatigue, injection site reactions)	Lower tolerability vs. RCTs; cost relevant	Kanaan et al. (2020)
Erenumab	Observational cohort RWE	Mostly CM, heavily pretreated (n = 101)	Observational	↓ headache days (−6.5); ↓ migraine days (−8.4)	28% discontinuation; constipation common	Wearing-off phenomenon reported	Robblee et al. (2020)
Erenumab	EARLY 2 (Italy)	HFEM + CM, ≥3 failures (n = 221)	Multicenter RWE; 48 weeks	≥50% response: 75.6% (CM), 56.1% (HFEM)	Good tolerability	Dose escalation often needed	Barbanti et al. (2021)
Erenumab	MAGIC study	EM + CM, ≥2 failures	Prospective observational; 24 weeks	∼33% ≥ 50% response; sustained	Well tolerated	Better outcomes in EM	Becker et al. (2022)
Erenumab	APOLLON study	EM + CM (n = 701)	Multicenter; 128 weeks	Sustained efficacy; ↓ disability	Discontinuation 4.1%; no new signals	Re-initiation restores response	Gobel et al. (2024)
Eptinezumab	FAERS pharmacovigilance	Spontaneous reports	Retrospective safety analysis	Ineffectiveness most reported	Signals: intracranial pressure, URTI; early AEs	Strong reporting bias; no denominator	Chen et al. (2025)
Eptinezumab	Mistry et al. systematic review	CM (7,352 patients)	51-study systematic review	Anti-CGRP mAbs most effective; Eptinezumab among best	Serious AEs rare; mostly mild	Higher cost but best QoL gain	Mistry et al. (2024)
Galcanezumab	Systematic RWE review	EM + CM	Systematic review	Comparable to RCTs; sustained benefit	Consistent safety profile	Confirms external validity	Pozo-Rosich et al. (2024)
Galcanezumab	MarketScan study	EM adults	Retrospective; 12 months	Higher persistence vs. rimegepant	No new safety signals	Lower cost increase	Kim et al. (2025)
Fremanezumab	Phase 3 randomized clinical trial	875 adults with episodic migraine	Randomized, placebo-controlled clinical trial; 12 weeks	Reduction in monthly migraine days *versus* placebo: −1.5 days with monthly dosing and −1.3 days with single high-dose regimen	Most common ADRs were injection-site reactions, and treatment discontinuation due to ADRs was ∼2% across groups	Demonstrated efficacy in episodic migraine	Dodick et al. (2018b)
Fremanezumab	Phase 3b randomized double-blind placebo-controlled study	838 patients (18–70 years) with episodic or chronic migraine and 2–4 previous preventive treatment failures	Multicenter phase 3b RCT; 104 centers in Europe and the US	Significant reduction in monthly migraine days: quarterly regimen −3.7 days/month; monthly regimen −4.1 days/month *versus* placebo	Serious adverse events (SAEs) were rare (∼1%) and similar between groups	Included difficult-to-treat population with prior preventive failures	Ferrari et al. (2019)
Fremanezumab	PEARL study protocol	Patients with episodic and chronic migraine from ∼100 centers across 11 European countries	Prospective real-world observational study; planned duration 24 months	Study designed to evaluate effectiveness, adherence, and persistence in clinical practice	Planned assessment of ADRs and long-term tolerability	Largest prospective European real-world study on Fremanezumab	Ashina et al. (2021a)
Fremanezumab	PEARL initial real-world results (Ashina et al., 2023)	Real-world migraine population treated in European centers	Prospective observational real-world analysis	Efficacy endpoints achieved in routine clinical practice	Approximately 17% of patients experienced >1 drug-related ADR; most frequent ADRs were injection-site reactions	Confirmed effectiveness and tolerability outside controlled trial settings	Ashina et al. (2023)
Fremanezumab	PEARL third interim analysis	968 patients with episodic or chronic migraine	Prospective real-world observational analysis; up to 12 months follow-up	58.5% achieved ≥50% reduction in monthly migraine days within 6 months	TRAEs were predominantly mild/moderate; injection-site reactions most common. Constipation reported in 3.9%	High adherence (≥90% up to month 12) and sustained long-term effectiveness and tolerability in real-world clinical practice	Ashina et al. (2025)

In the same year, another post-marketing observational study also reported constipation as one of the major adverse events, while confirming the good clinical efficacy of Erenumab. This study, conducted on 101 patients with migraine, mostly chronic and in a heavily pretreated population, showed that in the 43 patients with 6-month follow-up, the monthly days of headache and migraine were reduced by an average of 6.5 and 8.4 days, respectively. However, 28% of patients discontinued treatment, mainly due to adverse effects or ineffectiveness. In addition to constipation, a “wearing-off” effect was also reported in some cases (Tables 1, 6) (Robblee et al., 2020).

The Italian multicentre real-world study ‘EARLY 2’, conducted on 221 patients with high-frequency episodic migraine (HFEM) or CM evaluated the efficacy and tolerability of Erenumab for up to 48 weeks, offering a solid and clinically relevant long-term perspective. The data obtained in this study reflect the condition of a population resistant to standard treatments, as all patients had failed at least three previous preventive treatments. In this case too, the results showed a significant reduction in the number of days per month with migraine or headache, an improvement in QoL, and a marked decrease in the use of analgesics, especially in patients with chronic migraine. 75.6% of patients with CM and 56.1% with HFEM achieved at least a 50% reduction in migraine days. However, most patients required an increase in dose to 140 mg, suggesting that it may be useful to start directly with this dosage (Tables 1, 6) (Barbanti et al., 2021).

In this prospective, observational MAGIC study, evaluated the efficacy and safety of Erenumab in real-world clinical practice in Canada, in patients who mostly received the 140 mg monthly dose. In this case, the dose was chosen by the physician based on the severity of the clinical picture, and adult patients with episodic or chronic migraine who had already failed two or more preventive treatments were involved. After 3 months of treatment, approximately one-third of patients achieved a 50% or greater reduction in monthly migraine days, with slightly better results in patients with episodic migraines than in those with chronic migraines. At week 24, response rates remained stable. The treatment was well tolerated and safe, providing further real-world evidence to support the use of Erenumab in patients with a history of treatment failure. In fact, in this case too, all patients have already failed two or more categories of prophylactic therapies, making the population representative of complex and realistic clinical cases often observed in daily practice. In addition to subjective patient assessments collected via electronic diaries and questionnaires, the study also included clinical assessments by physicians, who observed improvements in over 75% of cases. This balanced approach between subjective perception and clinical observation is rare in real-world studies and increased the robustness of the data in this case. Finally, the 12- and 24-week follow-up allowed for the evaluation of both early and sustained response over time, providing a comprehensive view (Tables 1, 3) (Becker et al., 2022).

In 2024, a systematic review of real-world observational studies was published, examining 12 prospective cohort studies that included a total of 2,427 patients with episodic and chronic migraine. Overall, Erenumab was shown to be effective and generally safe, resulting in a clinically significant reduction in monthly migraine days and monthly headache days, and a reduced need for symptomatic medications to stop attacks (such as triptans or analgesics), indicating better disease control and a lower risk of medication overuse. The improvement in the overall impact of migraine on patients’ lives was manifested by less interference from migraine in daily activities; a reduction in disability and days lost from work or activities; and an improvement in physical, social, and emotional wellbeing. This analysis showed that a significant proportion of patients, including those who had been refractory to previous therapies, maintained their response over time, with frequent conversion from chronic to episodic migraine. The safety profile was therefore favourable, with predominantly mild adverse events and low discontinuation rates. Nisar et al. conclude this study by stating the need for further long-term studies to confirm their findings (Table 1) (Nisar et al., 2024).

In the same year, the multicentre APOLLON study evaluated the long-term (128 weeks) safety and tolerability, as well as the management of treatment discontinuation with Erenumab in 701 adult patients with episodic or chronic migraine. The results confirm a favourable safety profile consistent with previous clinical data, with no new safety signals and a low discontinuation rate due to adverse events (4.1%). Discontinuation of treatment was associated with a significant worsening of monthly migraine days and monthly headache days, as well as acute medication use, while resumption of Erenumab therapy resulted in rapid and substantial reversal of the worsening, with recovery of efficacy similar to initial treatment. These data reinforce the evidence on the long-term safety and therapeutic flexibility of Erenumab in clinical practice (Tables 1, 3) (Gobel et al., 2024).

### 3.2 Galcanezumab

The second mAb approved for the treatment of migraine was Galcanezumab, sold under the trade name Emgality. It is a humanised monoclonal antibody developed by Eli Lilly and Company specifically for the treatment of migraine in adults, approved in the United States in September 2018. The dosage is a single monthly dose of 120 mg (Urits et al., 2020).

Much information on the efficacy and safety of the monoclonal antibody Galcanezumab in the prophylaxis of episodic and chronic migraine has emerged from the phase 3 REGAIN and EVOLVE-1/EVOLVE-2 studies (Nichols et al., 2019).

The REGAIN study, comprising a 3-month randomised phase and a 9-month open-label extension, demonstrated that Galcanezumab (120 mg and 240 mg) significantly reduces monthly migraine days compared to placebo, with sustained and progressive benefits over time and no significant increase in adverse events. Efficacy was independent of previous treatment failures, with functional improvements and reduced use of abortive medications (Table 2) (Detke et al., 2018).

The EVOLVE-1 and EVOLVE-2 studies, conducted on over 1,700 patients with episodic migraine, confirmed a significant reduction in monthly headache days compared to placebo, with a good tolerability profile and no clinically relevant differences between the 120 mg and 240 mg doses. After discontinuation of treatment, efficacy was partially reduced but remained superior to placebo, suggesting a possible persistent effect.

Aggregate analyses also showed a similar benefit in patients with low- and high-frequency episodic migraine, with an overall reduction in migraine days of approximately 45%–50%, confirming Galcanezumab as an effective and well-tolerated option for migraine prevention. In fact, in these studies, the side effects observed were mostly mild or moderate and rarely led to discontinuation of treatment. The most frequently reported side effects were reactions at the injection site (pain, erythema, itching), upper respiratory tract infections, and mild skin reactions. In the EVOLVE-1 and EVOLVE-2 studies, the overall incidence of adverse events was less than 5%, and no significant differences were observed between the 120 mg and 240 mg doses. Furthermore, no serious adverse events related to treatment were observed (Tables 2, 4) (Silberstein et al., 2019).

**TABLE 4:** Efficacy and safety evidence of Galcanezumab. Summary of the main available evidence on the efficacy and safety of Galcanezumab from both clinical trials and real-world studies.

Study	Population	Design/Duration	Efficacy	Safety	Key notes	Ref.
REGAIN (Phase 3)	Chronic migraine	Randomized, double-blind (3 months) + open-label extension (9 months)	Significant reduction in MMD vs. placebo; sustained benefit; efficacy independent of prior failures; reduced acute medication use	No increase in AEs; well tolerated	Long-term efficacy maintained; effective in treatment-resistant patients	Detke et al. (2018)
EVOLVE-1 and EVOLVE-2 (Phase 3)	Episodic migraine (>1,700 patients)	Randomized, double-blind, placebo-controlled	Significant reduction in MHD vs. placebo; ∼45–50% reduction; consistent across EM subtypes; partial persistence after discontinuation	Mostly mild/moderate AEs; <5% incidence; no dose-related differences; no serious treatment-related AEs	No added benefit of 240 mg vs. 120 mg	Silberstein et al. (2019)
Pooled analyses	Episodic migraine (low/high frequency)	Post hoc pooled analysis	Consistent ∼45–50% reduction in migraine days across subgroups	Safety consistent with RCTs	Confirms efficacy across EM spectrum	Pozo-Rosich et al. (2024)
Real-world evidence (systematic review)	Episodic and chronic migraine	Systematic review of observational studies	Effectiveness comparable to RCTs	Safety consistent with trials	Supports generalizability to clinical practice	Pozo-Rosich et al. (2024)
MarketScan study (2020–2023)	Episodic migraine (adults)	Retrospective cohort; 12-month follow-up; propensity-matched	Greater treatment persistence vs. rimegepant; lower discontinuation	Not primary focus; no new safety signals	Lower cost increase; improved treatment continuity	Kim et al. (2025)

More recently, a systematic review by Pozo Rosich et al. summarized real-world data from several prospective observational studies on the use of Galcanezumab in the prevention of episodic and chronic migraine. As already mentioned in other sections, real-world observations allow for the evaluation of aspects that cannot be fully explored in phase 3 studies. However, this review revealed results consistent with those reported in randomized trials, showing an overall comparable efficacy and safety profile and helping to better define the use of Galcanezumab in everyday clinical practice (Tables 3, 4) (Pozo-Rosich et al., 2024).

In addition, a retrospective study based on Merative™ MarketScan® databases (2020–2023) and published last year compared the use of Galcanezumab versus Rimegepant in adult patients with episodic migraine in terms of healthcare resources, costs and treatment persistence. After matching propensity scores, both cohorts showed an increase in total and migraine-related costs at 12 months; however, these increases were significantly lower in the Galcanezumab-treated group. An important factor was treatment persistence, which was higher with Galcanezumab, with a longer median time to discontinuation and a significantly lower discontinuation rate compared with Rimegepant. These results suggest significant differences in healthcare costs and treatment continuity between the two preventive strategies (Tables 3, 4) (Kim et al., 2025).

In summary, studies confirm that Galcanezumab is well tolerated, with a side effect profile limited mainly to local reactions and mild disorders, consistent with what is expected for anti-CGRP monoclonal antibodies.

### 3.3 Fremanezumab

In the same year that Erenumab and Galcanezumab were approved, a randomized clinical trial was published examining the efficacy of a fully humanized anti-CGRP antibody: Fremanezumab. In this study involving 875 adults with episodic migraine (with no previous failures with multiple classes of drugs), Fremanezumab was found to lead to a significant reduction in monthly migraine days compared to placebo. The reduction was 1.5 days with monthly dosing and 1.3 days with a single high dose at baseline, over a period of 12 weeks. In addition to verifying the efficacy of Fremanezumab, this study also examined adverse events and found that they most commonly affected the injection site (erythema, induration, etc.). The percentage of patients who discontinued treatment due to adverse events was similar in each treatment group (2%). However, the authors of this study emphasized the need for further research to compare the efficacy of Fremanezumab with other preventive drugs, to evaluate its efficacy in patients with previous treatment failures, and to determine its long-term safety and efficacy (Tables 2, 3, 5) (Dodick et al., 2018b).

**TABLE 5:** Efficacy and safety evidence of Fremanezumab. Summary of the main available evidence on the efficacy and safety of Fremanezumab from both clinical trials and real-world studies.

Study	Population	Design/Duration	Efficacy	Safety	Key notes	Ref.
Phase 3 RCT (episodic migraine)	Episodic migraine (n = 875; no multiple prior treatment failures)	Randomized, double-blind, placebo-controlled; 12 weeks	Reduction in MMD vs. placebo (−1.5 days monthly; −1.3 days single high dose)	Injection site reactions most common; ∼2% discontinuation; similar across groups	First evidence of efficacy; limited data in refractory patients	Dodick et al. (2018b)
Phase 3b RCT (treatment-resistant migraine)	Episodic/chronic migraine with 2–4 prior preventive failures (n = 838)	Randomized, double-blind, placebo-controlled; monthly vs. quarterly dosing	Significant reduction in MMD vs. placebo (−3.7 to −4.1 vs. −0.6 days)	Similar AEs across groups; serious AEs ∼1%	Confirms efficacy in difficult-to-treat population; supports flexible dosing	Ferrari et al. (2019)
PEARL study (real-world)	Episodic and chronic migraine	Prospective observational; up to 24 months (interim analyses at 6–12 months)	≥50% responder rate 58.5% at 6 months; sustained MMD reduction; reduced acute medication use and disability	Mostly mild/moderate AEs; injection site reactions most common; ∼17% ≥ 1 ADR; no new safety signals	Largest prospective real-world study; higher responder rates vs. RCTs	Ashina et al. (2021b); Ashina et al. (2023)
PEARL study (12-month analysis)	Real-world migraine population	Prospective observational; 12-month follow-up	Early and sustained reduction in migraine frequency, severity, and duration	Constipation 3.9%; overall favorable tolerability	High adherence (≥90%) and persistence; strong external validity	Ashina et al. (2025)

The following year, another clinical study was published on the effects of Fremanezumab in migraine patients who had not responded to two to four classes of preventive drugs. Again, the efficacy of this biological drug was tested and confirmed in terms of reducing the number of migraine days and the use of other acute-phase medications. Regarding adverse reactions to the drug, the data were comparable across all groups. Specifically, this was a phase 3b, randomized, double-blind, placebo-controlled study. The study collected data from 104 centers in various European countries and the US and included 838 patients aged between 18 and 70 years with episodic or chronic migraine and a history of two to four previous preventive treatment failures in the last 10 years due to ineffectiveness, intolerance, or contraindications. The study involved randomization into three groups: (i) quarterly Fremanezumab; (ii) monthly Fremanezumab; (iii) monthly placebo and aimed to determine the mean change in the number of monthly migraine days from baseline. In this case, too significant improvements were observed compared to placebo (quarterly Fremanezumab: –3.7 days/month (vs. placebo −0.6 days); monthly Fremanezumab: –4.1 days/month; difference: –3.5 days). Side effects were similar in the three groups, and serious adverse events were rare and similar across groups: approximately 1% (Tables 2, 3, 5) (Ferrari et al., 2019).

In 2021, the protocol for the real-world study “Pan-European Real Life” (PEARL) was published: a 24-month prospective observational study on the effects of Fremanezumab, which would examine data from around 100 centers across 11 European countries (Tables 3, 5) (Ashina et al., 2021b). The authors of this study analyzed efficacy, adherence, persistence, and ADRs during treatment. The initial results of this study were published in 2023. The efficacy endpoints were achieved as efficacy was demonstrated. Concerning adverse events, approximately 17% of patients experienced more than one drug-related adverse event, and again, the most common adverse events were related to the injection site (Table 5) (Ashina et al., 2023). The third interim analysis of the PEARL study evaluated the long-term efficacy and safety of Fremanezumab in the prevention of episodic and chronic migraine for up to 12 months of treatment. Of the 968 patients included in the efficacy analysis, 58.5% achieved a ≥50% reduction in monthly migraine days within the first 6 months of treatment, with benefits maintained up to 12 months, a higher percentage than that reported in randomized controlled trials (38%–47%). Over the course of 12 months, sustained reductions in monthly migraine days, acute medication use, and disability scores (MIDAS and HIT-6) were observed, with no new safety signals emerging. These real-world data support the long-term efficacy and tolerability of Fremanezumab in a broad population representative of clinical practice. The PEARL study, the largest prospective real-world study on Fremanezumab, had the advantage of evaluating a large and heterogeneous population of patients with episodic and chronic migraine. Another finding was a reduction in the severity and duration of migraine attacks, which was already evident within the first month of treatment and was maintained over time. Adverse events related to treatment were predominantly mild or moderate, with injection site reactions being the most common side effects. Constipation was reported in 3.9% of participants, a lower percentage than that reported for other anti-CGRP treatments, particularly those targeting the CGRP receptor. Adherence and persistence data showed encouraging results: most patients continued treatment for up to 12 months, and adherence rates were ≥90% for all injections up to month 12. Overall, despite the inherent limitations of real-world studies (self-reported data and missing data), the PEARL study provided robust evidence of clinical efficacy, good tolerability, and high persistence for Fremanezumab in clinical practice, complementing and reinforcing the results of randomised trials (Tables 3, 5) (Ashina et al., 2025).

### 3.4 Eptinezumab

Eptinezumab (Vyepti™, Lundbeck Seattle BioPharmaceuticals, Inc., Bothell, WA, United States) is a humanized anti-CGRP monoclonal antibody recently approved by the US Food and Drug Administration (FDA) for the preventive treatment of migraine in adults. It received marketing authorization in the European Union, including Italy, following approval by the EMA in 2022. It is administered intravenously, allowing therapeutic concentrations to be reached quickly (Silberstein et al., 2020).

In pivotal Phase 3 PROMISE (PRevention Of Migraine via Intravenous ALD403 Safety and Efficacy) clinical trials, conducted in patients with episodic and chronic migraine, the 100 mg and 300 mg doses achieved the primary efficacy endpoints, demonstrating a favorable safety profile, with treatment-emergent adverse events predominantly mild or moderate in severity (Tables 2, 6) (Ashina et al., 2020).

**TABLE 6:** Efficacy and safety evidence of Eptinezumab. Summary of the main available evidence on the efficacy and safety of Eptinezumab from both clinical trials and real-world studies.

Study	Population	Design/Duration	Efficacy	Safety	Key notes	Ref.
PROMISE program (Phase 3 RCTs)	Episodic and chronic migraine	Randomized, double-blind, placebo-controlled	Both 100 mg and 300 mg achieved primary endpoints; significant reduction in MMD	TEAEs mostly mild/moderate; favorable safety profile	IV administration allows rapid onset; consistent efficacy across populations	Ashina et al. (2020)
PROMISE-2 (Phase 3 RCT, chronic migraine)	Chronic migraine (n = 1,072)	Randomized, double-blind, placebo-controlled; IV dosing at baseline and week 12; 24-week follow-up	Significant MMD reduction vs. placebo; sustained benefit; higher ≥50% and ≥75% responder rates; improved HIT-6 and PGIC scores	No new safety signals; AEs consistent with prior data	Demonstrates sustained efficacy with repeated dosing	Lipto et al. (2020)
FAERS pharmacovigilance analysis (real-world)	Real-world population (spontaneous reports)	Retrospective database analysis	Reports of perceived ineffectiveness; limited direct efficacy assessment	Signals: intracranial pressure, URTI; early onset AEs; higher reporting in women; increased vulnerability in elderly (≥65 years)	Highlights need for monitoring; limitations due to reporting bias and lack of denominator	Chen et al. (2025)
Systematic review (Mistry et al.)	Chronic migraine (7,352 adults)	Systematic review of 51 studies	Anti-CGRP mAbs most effective in reducing MMD/MHD; superior QoL vs. topiramate and onabotulinumtoxinA; Eptinezumab among most effective	Serious AEs rare; overall favorable safety	High efficacy but higher cost; favorable cost-effectiveness profile	Mistry et al. (2024)

In this context, the phase 3, randomized, double-blind, placebo-controlled PROMISE-2 study evaluated the efficacy and safety of repeated doses of Eptinezumab in the prevention of chronic migraine. The study included 1,072 adults treated with Eptinezumab 100 mg, 300 mg, or placebo administered intravenously on day 0 and week 12. Both doses of Eptinezumab resulted in a significantly greater reduction in monthly migraine days compared to placebo, with further improvement after the second administration and a sustained benefit for 24 weeks. Response rates of ≥50% and ≥75%, as well as improvements in patient-reported outcomes (Headache Impact Test and Patient Global Impression of Change), were consistently higher in the Eptinezumab groups than in the placebo group. From a safety perspective, no new signals emerged: the incidence, nature, and severity of adverse events were consistent with what had been previously reported. Therefore, 100 mg and 300 mg of Eptinezumab administered intravenously demonstrated a lasting preventive benefit and an acceptable safety profile in patients with chronic migraine (Tables 2, 6) (Lipto et al., 2020).

Eptinezumab shows promise, but despite the information already acquired from clinical trials, further research is needed in real-world settings to investigate its safety profile in vulnerable populations.

Some information in this context has been obtained from pharmacovigilance analysis using data from the FDA Adverse Event Reporting System (FAERS) database. This publicly accessible database collects information from a spontaneous reporting system for reports from consumers, doctors, and pharmacists. The aim of this analysis was therefore to identify emerging safety signals and better understand the profile of adverse events (AEs) in the real world. Among the most frequently reported events was the ineffectiveness of the drug, but associations with increased intracranial pressure and upper respiratory tract infections were also observed, suggesting that CGRP inhibition may affect central nervous system function and immune response. The analysis also found differences by sex and age. Women reported more adverse events than men, probably due to the higher prevalence of migraine and biological and behavioural factors, while in elderly patients (aged 65–85 years) there were stronger signs of therapeutic ineffectiveness and some rare serious adverse events, such as facial paralysis. These results indicate greater vulnerability in elderly populations and highlight the need for careful monitoring. This analysis also highlighted a temporal cluster of early events, especially during the first month of treatment, suggesting that adverse reactions tend to occur in the early stages and emphasizing the importance of close monitoring during this period. Finally, the analysis highlighted the complexity of interpreting safety signals due to under-reporting, reporting bias, and incomplete patient information, which are typical of spontaneous pharmacovigilance databases. These data indicate the need to integrate real-world information with clinical studies for a more comprehensive understanding of risks, particularly for serious or rare adverse events, and to optimize personalized treatment strategies, not least because an intrinsic limitation of the FAERS database was the inability to know the total number of patients prescribed Eptinezumab (Tables 3, 6) (Chen et al., 2025).

In a recent literature review, Mistry et al. analyzed 51 articles containing data on the treatment of chronic migraine in 7,352 adults. This analysis compared six drugs (anti-CGRP monoclonal antibodies, Botox and topiramate) with a placebo. Again, mAbs were shown to reduce the number of monthly headache/migraine days, and the reduction was greater than that achieved with the other drugs under review. Anti-CGRP drugs also showed the best results in terms of QoL, while topiramate was less effective than anti-CGRP drugs and Botox. This systematic review also showed that Eptinezumab and Fremanezumab administered monthly were the most effective.

In line with other studies, serious adverse events during mAb therapy were rare; the most common reactions included are at the injection site. From an economic point of view, injectable anti-CGRP drugs were found to be more expensive but generally had a good cost-effectiveness profile. In fact, topiramate was the least expensive option but offered the least benefit in terms of QoL, while Eptinezumab 300 mg offered the greatest benefit but at the highest cost (Tables 3, 6) (Mistry et al., 2024).

## 4 Conclusion

Real-world clinical evidence provides an opportunity to redefine the benefit-risk profile and value of biotech medicines in the general population. To better characterize the tolerability profile of biosimilars, post-marketing surveillance activities need to be implemented. In this context, practical and rational use of biosimilars plays an important role in the financial sustainability of regional healthcare systems. Recently, some real-world studies comparing various anti-CGRP drugs have also begun to emerge. For example, Sun et al. used the FDA database to observe the most common AEs observed with the four biologics studied. The most frequent adverse effect was irritation at the injection site, in line with what had already been observed in clinical trials. However, thanks to this study, new adverse events not previously mentioned have been added to the package leaflet, such as menstrual disorders, Raynaud’s phenomenon, weight gain, throat tightness, and oral paraesthesia (verified simultaneously with multiple drugs) (Sun et al., 2023). In addition to injection-site reactions, emerging pharmacovigilance data have highlighted the need for continued monitoring of cardiovascular and vascular safety signals potentially associated with CGRP blockade, particularly in patients with pre-existing vascular risk factors (de Vries Lentsch et al., 2021; Ornello et al., 2020). Although current evidence does not indicate a major increase in serious cardiovascular adverse events, the physiological role of CGRP in vasodilation and vascular homeostasis warrants long-term surveillance in real-world populations. Furthermore, post-marketing studies have emphasized the importance of monitoring less represented populations, including elderly patients, individuals with multiple comorbidities, and patients receiving concomitant therapies, who are often underrepresented in randomized clinical trials (Robbins, 2020).

Therefore, this review collects several pieces of evidence on the concept that real-world studies contribute to our understanding of the safety of anti-CGRP monoclonal antibodies in clinical settings and provide valuable information on the clinical selection of drugs. Another important aspect that remains to be clarified is the safety profile of these drugs during pregnancy. In fact, during pregnancy, systemic CGRP levels increase, contributing to vascular adaptations; therefore, guidelines suggest avoiding the use of anti-CGRP antibodies during pregnancy, even though no specific toxicities have been identified. Due to ethical restrictions, most information on drug safety during pregnancy comes from post-marketing studies. However, before determining the safety of Erenumab, Galcanezumab, and Fremanezumab in pregnancy, sufficiently representative databases are needed. Similarly, additional evidence is needed regarding long-term exposure and safety in special populations, including pregnant women, older adults, and patients with cardiovascular or autoimmune disorders. International pregnancy registries, active pharmacovigilance programs, and real-world longitudinal cohort studies will therefore be essential to better define the long-term safety profile of anti-CGRP therapies (de Vries Lentsch et al., 2021; Noseda et al., 2023).

Nevertheless, pharmacovigilance is expected to continue to evolve and monitoring to improve technology is expected to support this development. Social media surveillance will become routine. Increasingly large and diverse databases will be regularly linked. Pharmacovigilance will make progress in mitigating the consequences of errors. All rapidly evolving technologies will lead to faster and wider adoption by professionals. In this evolving scenario, collaboration between clinicians, pharmacovigilance networks, and regulatory agencies will be crucial to ensure early detection of rare adverse events, improve traceability, and optimize benefit-risk assessment in real-world settings. The integration of artificial intelligence tools, electronic health records, and large pharmacovigilance databases may further enhance signal detection and support personalized therapeutic decision-making (Salas et al., 2022).

The available results show that the efficacy and safety data obtained from clinical and real-life studies on anti-CGRP antibodies are comparable. However, the observation needs to be extended to other less-represented segments of the population. Despite the overall consistency between randomized clinical trials and real-world evidence, the interpretation of currently available data requires caution. Many observational and pharmacovigilance studies are intrinsically limited by potential reporting bias, incomplete clinical information, heterogeneous populations, and the lack of standardized control groups. In addition, spontaneous reporting databases cannot establish causal relationships or accurately estimate the incidence of adverse events. These limitations should be considered when interpreting the reassuring safety profile emerging from current evidence, particularly for rare adverse events and long-term outcomes in vulnerable or underrepresented populations.
